# The regulatory interplay between Oct-1 isoforms contributes to hematopoiesis and the isoforms imbalance correlates with a malignant transformation of B cells

**DOI:** 10.18632/oncotarget.25648

**Published:** 2018-07-06

**Authors:** Elizaveta V. Pankratova, Alexander G. Stepchenko, Irina D. Krylova, Tatiana N. Portseva, Sofia G. Georgieva

**Affiliations:** ^1^ Engelhardt Institute of Molecular Biology, Russian Academy of Sciences, Moscow, Russia; ^2^ Pirogov Russian National Research Medical University, Moscow, Russia

**Keywords:** POU2F1, isoforms, hematopoietic cells differentiation, molecular therapeutic target

## Abstract

Oct-1(POU2F1) is a DNA-binding transcription regulator and its level being highly increased in many human cancers. Oct-1 is present in the human cells as a family of functionally different isoforms which are transcribed from alternative promoters. Here, we have demonstrated that expression patterns of Oct-1 isoforms change during differentiation of hematopoetic progenitor cells (CD34+) (HPCs) to the B (CD19+) and T (CD3+) cells. While Oct-1L is expressed at a high level in the CD34+ HPCs, its expression level drops dramatically during the T-cell differentiation, although remains nearly the same in B-cells. We have described the novel human Oct-1R isoform which is conserved in mammals and is B cell-specific. Oct-1R was found in B cells, but not in HPCs. Oct-1R is transcribed from the same promoter as Oct-1L, another lymphocyte-specific isoform. Overexpression of Oct-1R and Oct-1L in the Namalwa cells leads to the repression of many genes involved in B-lymphocyte differentiation and signal transduction. Thus these isoforms may regulate the particular stages of development of normal B cells and maintain their proper differentiation status. However the extremely high level of Oct-1L isoform observed in the B-lymphoblast tumor cell lines indicated that the excess of Oct-L seem likely to considerably decrease the differentiation ability of these cells. Oct-1 may serve as a therapeutic target for many tumors, but it should be noted that in a tumor the content of a certain isoform Oct-1, rather than the total Oct-1 protein, can be increased.

## INTRODUCTION

Oct-1(POU2F1), the member of the group of DNA-binding transcription regulators of higher eukaryotes, controls a wide range of targets including the housekeeping genes and genes specific for immune, endocrine, and nervous systems [1–15]. On the basis of the amino acid sequences of their DNA-binding domains, Oct-1 together with three other proteins [Pit1, Oct-2, and Unc-86) were originally assigned to the family of the POU-domain transcription factors [1–3]. Later POU domain was found in a number of other transcription factors including the stem cell marker Oct-4.

The ubiquitous expression and the presence of Oct-1 on the promoters of the H2B gene [1] and the genes of snRNAs U2, U6, and 7SK [5–7] provided grounds to the general concept that Oct-1 regulates the expression of housekeeping genes. The later works have, however, demonstrated that the function of Oct-1 is broader and much more sophisticated, than it was previously expected. In particular, Oct-1 controls the expression of a considerable number of tissue-specific genes. It plays a significant role in the immune system development and function being the expression regulator of the IL2 [8], IL8 [9], IL3, and IL5 interleukin genes, GCSF gene [13], mb-1 [11] and B29 [12], Ly9 [13], and the genes encoding light and heavy immunoglobulin chains [14]. It also regulates the expression of the endocrine system genes including the genes encoding Pit-1 [16], gonadotropin releasing hormone [17], prolactin [18], thyroid transcription factor [19], and thyrotropin [25]. Oct-1 may function as both the transcription activator and repressor. It has been estimated that about 2500 genes in the human genome are the targets for Oct-1 (http://www.genecards.org). In addition, Oct-1 was shown to participate in the replication of adenovirus, and probably also eukaryotic, genomes [20, 21], organization of the matrix attachment regions (MARs) [22], and interaction with HMG proteins [23]. Oct-1 is essential for the control of apoptosis [24–26] and glycolytic metabolism [24, 27].

Oct-1 is co-expressed with Oct-4 in embryonic cells and is essential for the differentiation of embryonic stem cells being a key mediator of the development-specific gene activation and repression [28]. In the Oct-1−/− loss of function model, early embryonic lethality was observed [29]. High levels of Oct-1 were detected in somatic stem cells and cancer stem cells and correlated with tumor aggressiveness in gastric, prostate, cervical, head and neck cancer, esophageal, colorectal cancers [30–35].

Hence, the actual diversity of Oct-1 functions is exceptionally high given that it controls the expression of both the housekeeping genes and the genes which are highly specific for certain tissues, as for example, Pit1 whose expression is restricted to the cells of adenohypophysis, or immunoglobulin genes which are expressed in B-lymphocytes and plasma cells. Several factors contribute to the polyfunctionality of Oct-1. One of them is the plasticity of POU domain which can bind target DNA sequences of different structure either as monomer, dimer, or heterodimer [7, 9, 16, 36–41]. Oct-1 is also able to interact with a broad number of proteins involved in the transcription regulation. In addition, Oct-1 undergoes multiple posttranslational modifications including phosphorylation, O-glycosilation, and sumoylation [42–48], which modulate the specificity of its interaction with the target genes.

The important factor of Oct-1 polyfunctionality is that it exists in the cell in a number of different isoforms which constitute the family of Oct-1 factors. At present time, 7 RefSeq and 14 additional Oct-1 mRNA sequences have been identified in humans (http://www.genecards.org). Multiple alternative isoforms of Oct-1 have been reported in mouse cells [49–52]. Interestingly, a large part of Oct-1 isoforms are conserved between the two species, which implies the importance of their functions.

In the previous works, we have demonstrated the existence of three alternative promoters (U, L, and X) in the human *POU2F1* gene [25, 50, 53–56]. The corresponding transcripts have different first exons and encode Oct-1A, Oct-1L, and Oct-1X isoforms, respectively, which differ in their N-terminal sequences [25].

We have demonstrated that the longest isoform, Oct-1A, is abundantly expressed and represents the main Oct-1 protein in most human tissues. The Oct-1L is expressed at a rather low level in several tissues including blood cells and brain, with the highest levels of its expression being observed in B-cells [25, 50]. Interestingly, we observed that the level of Oct-1L isoforms is increased in several types of tumor cell lines [54]. Oct-1X is expressed in a wide range of tissues but at low levels [25]. We have demonstrated that Oct-1L and Oct-1X regulate the major part of Oct-1A targets along with the sets of the isoform-specific genes, and also have several specific functions. Hence, the variation in the N-terminal part structure results in the difference in the patterns of genes regulated by different isoforms [25].

Here, we describe the new human isoform Oct-1R whose transcription starts at the L promoter and which is similar to Oct-1L with the exception of having a truncated C-terminus. Oct-1R expression is B cell-specific. A thorough analysis of the Oct-1 expression revealed that hematopoietic cell differentiation is associated with the significant changes in the expression patterns of Oct-1 isoforms. For example, while Oct-1L is expressed at a high level in the CD34+ hematopoietic progenitor cells (HPCs), its expression level drops dramatically during the T-cell differentiation, although remains nearly the same in B-cells. Oct-1R was found in B cells, but not in HPCs. Overexpression of Oct-1 isoforms in the Namalwa Burkitt lymphoma cell line and the functional enrichment analysis of differentially expressed genes (DEGs) performed here for the Oct-1R and previously for the Oct-1A,L,X isoforms [25] have demonstrated that there are both similarities and significant differences in the gene expression patterns for these isoforms. The most similar DEGs were revealed for Oct-1R и Oct-1L. Oct-1R represses a considerable number of genes responsible for B-cell differentiation and the regulation of immune response and signal transduction. Interestingly, the activity of the L promoter is lower than the activity of the U promoter in all normal hematopoietic cells, but significantly exceeds it in the B-cell lymphoblastoma lines Namalwa and Raji. Thus, the changes in the composition and relative ratios of the Oct-1 isoforms lead to the changes in the expression patterns of genes regulated by Oct-1 and in such a way the regulatory interplay between the Oct-1 isoforms contributes to cell differentiation.

## RESULTS

### Oct-1R isoform differs from Oct-1L isoform by the absence of 132 C-terminal amino acid residues and is specifically expressed in B-cells

**Figure 1:**
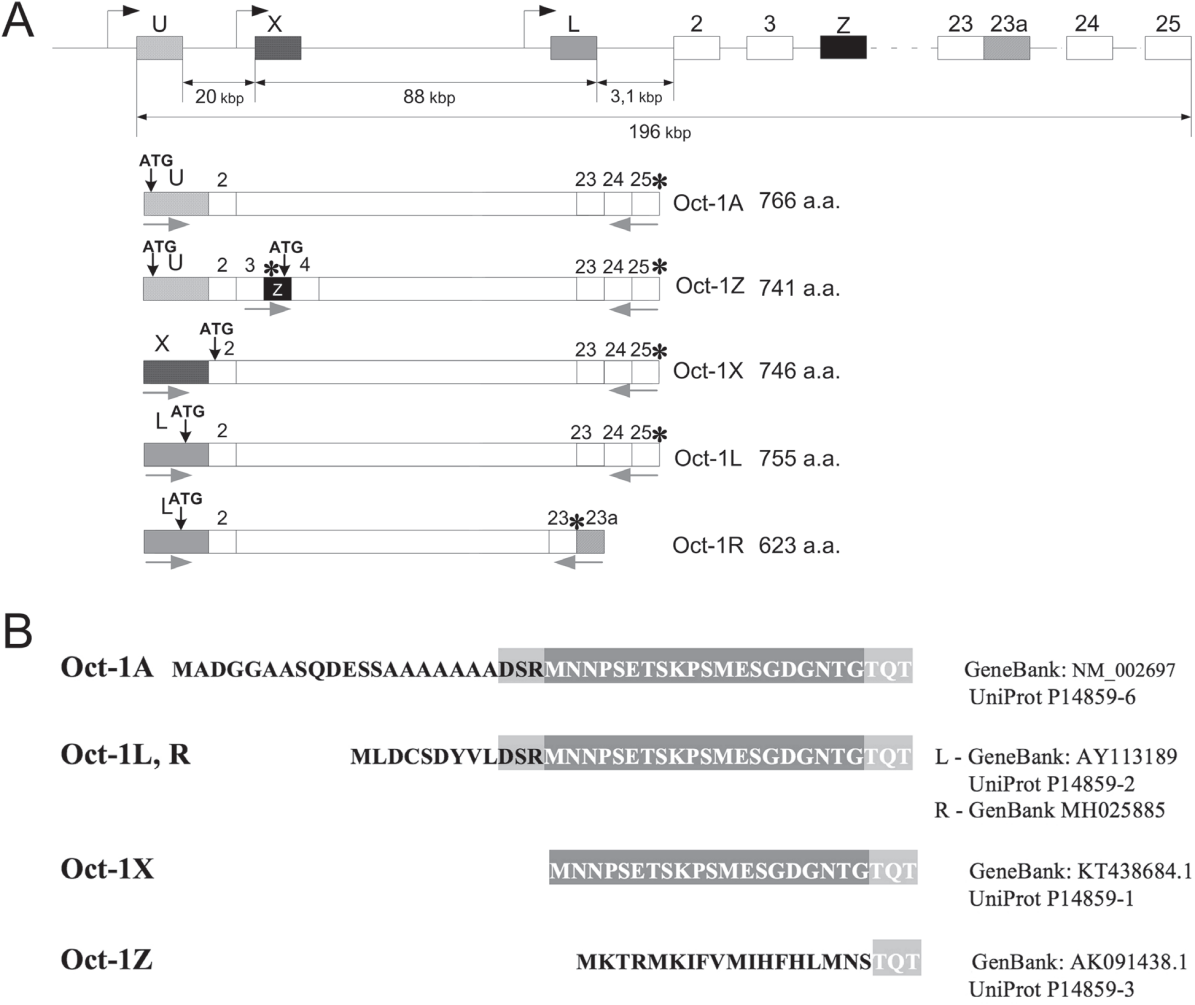
Schematic representation of the Oct-1 gene and its transcripts **(A)** Scheme of Oct-1 alternative promoters and Oct-1A, Oct-1L, Oct-1X, and Oct-1Z transcripts with different 5′-terminal exons. Oct-1R transcript has the additional 23a exon containing a stop codon. Alternative exons are shown as black or gray boxes. Transcription and translation starts are indicated by black arrows. Stop codons are indicated by asterisks. The positions of PCR primers are indicated with gray arrows. **(B)** Amino acid sequences of the N-terminal domains of Oct-1 isoforms. It should be noted that Oct-1L and Oct-1R isoforms have the same N-terminal region which differs from that of other isoforms.

Three alternative promoters U, L, and X for the human Oct-1 gene (Figure 1A) were characterized in our previous studies [25]. The resulting transcripts differ in their first exons and the corresponding Oct-1A, Oct-1L, and Oct-1X proteins have different N-terminal sequences (Figure 1B).

In the present work, we have cloned the new human Oct-1 transcript encoding the Oct-1R isoform (GenBank MH025885). Human Oct-1R transcript was obtained from the Burkitt lymphoma cell line. The transcript starts at the tissue-specific L promoter and the resulting Oct-1R isoform shares the N-terminal sequences and the intact POU domain with Oct-1L, while lacking 132 amino acid residues at the C-terminus due to the incorporation of the alternative exon 23a containing a stop codon (Figure 1A). So the presumable Oct-1R protein is the truncated form of Oct-1L from which 132 amino acid residues were trimmed at the C-terminus. We have previously described a similar isoform in mice [49].

**Figure 2:**
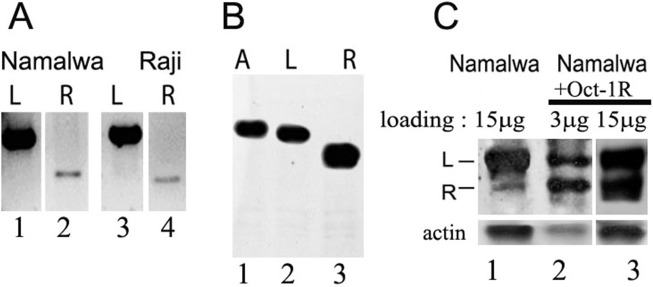
Oct-1R isoform transcription and translation **(A)** The full length Oct-1L and Oct-1R RNAs from Namalva (lanes 1 and 2) and Raji (lanes 3 and 4) cell lines obtained by RT-PCR and resolved on 1% agarose gel. **(B)** *in vitro* translation of Oct-1A (lane 1), Oct-1L (lane 2), and Oct-1R (lane 3) ORFs cloned in the pcDNA3.1 vector verified by Westen-blot. 1 μg of Oct-1 DNA was taken in each reaction. Western-blot was probed with anti-FLAG antibodies. **(C)** Western-blot of the protein extracts from the native Namalwa cells (15 μg of extract, lane 1) and the extracts from the Namalwa cells transformed with FLAG- tagged Oct-1R (Namalwa +Oct-1R) probed with antibodies against the anti-N-terminal peptide, specific for Oct-1L and Oct-1R isoforms. Two different concentrations of the Namalwa +Oct-1R cell extract (3 μg of extract, lane 2, or 15 μg of extract, lane 3) are used to better visualize the recombinant Oct-1R and to compare it with the endogenous isoform. The level of Oct-1R isoform was approximately estimated as 5% of the Oct-1L isoform level in Namalva cells (lane 1) using Amersham TM ECL TM Prime Western Blotting Detection Reagent and BIO-RAD ChemiDoc TM MP Imaging System Model. Actin expression was used as a loading control (lower panel).

The presence of the Oct-1R mRNA in various human tissues was tested by RT PCR. Different human tissues including brain, liver, lung, kidney, spleen, thymus, small intestine, kidney, testes, ovary, adipose, colon, esophagus, lung, bladder, kidney, cervix, heart, thyroid, trachea, prostate, placenta, skeletal muscles, as well as cell lines (Namalva and Raji B-cell lines, Jurkat T-cell line, MCF7, HeLa, and SKBR3) were examined. We detected the corresponding transcript only in the B-cell lines Namalva and Raji, which indicated that Oct-1R mRNA is most probably B-cell specific (Figure 2A, lanes 2 and 4).

This finding correlates well with the data obtained in mice experiments, where Oct-1R expression was found to be restricted to normal B lymphocytes and the NS/0 B-cell line [50]. Therefore, the two Oct-1 transcripts containing exon 1L (Oct-1L and Oct-1R) are expressed in B lymphocytes in addition to the ubiquitously expressed Oct-1A.

To demonstrate that Oct-1R protein is indeed synthesized, we first performed the *in vitro* transcription-translation of the cloned Oct-1R cDNA (Figure 2B). Oct-1R mRNA is efficiently translated *in vitro* (Figure 2B, lane 3). The molecular weight of the resultant protein corresponded well to the calculated molecular weight for Oct-1R. We have also detected Oct-1R in the Namalva cell extracts by Western blot (Figure 2C) using the previously obtained antibodies against the specific N-terminal peptide [25]. As it can be seen in Figure 1C (lane 1), the amount of the Oct-1R protein isoform in the extract is indeed significantly lower than that of the Oct-1L isoform, and was approximately estimated as 5% (see Figure legend) which correlates well with the transcription levels for the corresponding mRNAs. Molecular weight of the endogenous Oct-1R protein corresponds to that of the Oct-1R protein overexpressed in the Namalva cells (Figure 2C, Namalwa +Oct-1R, lane 2). The level of Oct-1L expression did not change in the Namalva cells transformed with Oct-1R (Figure 2C, compare lanes 1 and 3).

### The expression pattern of the human Oct-1 isoforms changes during the differentiation of hematopoietic cells

**Figure 3:**
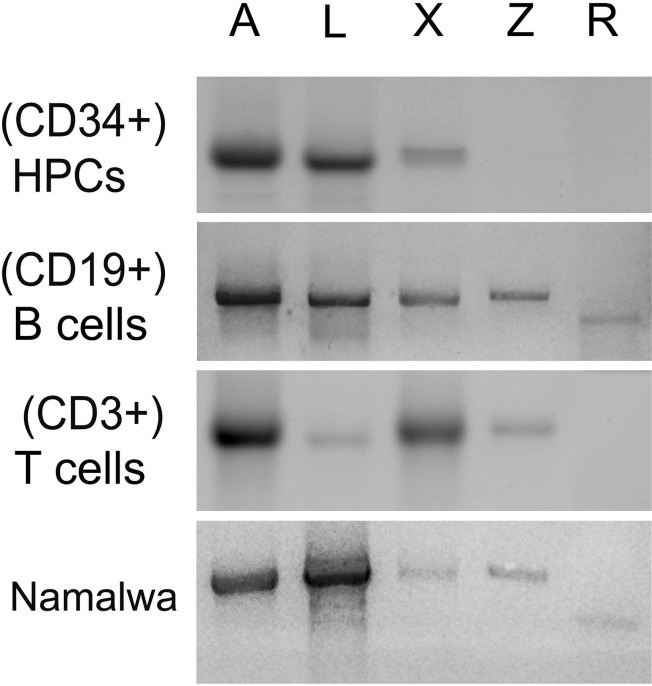
Oct-1 isoforms transcription Full length transcripts encoding Oct-1 isoforms in the human HPCs (CD34+), B cells (CD19+), T cells (CD3+), and in the human Namalwa Burkitt lymphoma cell detected by RT-PCR. The products of reaction were loaded on 1% agarose gel and stained with ethidium bromide. The isoforms are indicated above the figure: lane 1 (Oct-1A), lane 2 (Oct-1L), lane 3 (Oct-1X), lane 4 (Oct-1Z), and lane 5 (Oct-1R) isoforms.

We have analyzed the presence of five Oct-1 isoforms (Figure 1) in the CD34+ hematopoietic progenitor cells (HPCs) and in the B-cell (CD19+) and T-cell (CD3+) populations of peripheral blood. The mRNA levels for the Oct-1 isoforms were estimated by RT-PCR using primers which allowed detecting full-length transcripts (Figure 3). CD34+ HPCs were revealed to contain high levels of Oct-1 A (lane 1), L (lane 2), and X (lane 3) mRNAs. In the populations of CD19+ B-cells or CD3+ T-cells, mRNA levels for the ubiquitous Oct-1A isoform (lane 1) did not change, while the expression levels for other isoforms showed significant changes. Oct-1L mRNA level (lane 2), strongly decreased in the T cell population although it remained high in B-cells. The expression of Oct-1X (lane 3) significantly increased in B and T-lymphocytes. In both cell populations, we observed the activation of Oct-1Z expression (lane 4), while Oct-1R (lane 5) expression was detected only in the B-cell population. Interestingly, Z and R isoforms, which are the products of alternative splicing, are expressed only at later differentiation stages.

Thus, hematopoietic cell differentiation is associated not only with the high level of Oct-1 expression, but also with the significant changes in the expression patterns of Oct-1 isoforms.

### Increased transcription from the L promoter was observed in the tumor cell lines of B-cell origin compared to the normal B cells

We also assessed the expression levels for the Oct-1 isoforms in the Namalva Burkitt lymphoblastoma (Figure 3, the lower panel). Noteworthy, the level of the Oct-1L mRNA (lane 2) but not of other isoforms was strongly increased in the cancer cell line and significantly exceeded the level of the Oct-1A isoform (lane 1).

**Figure 4:**
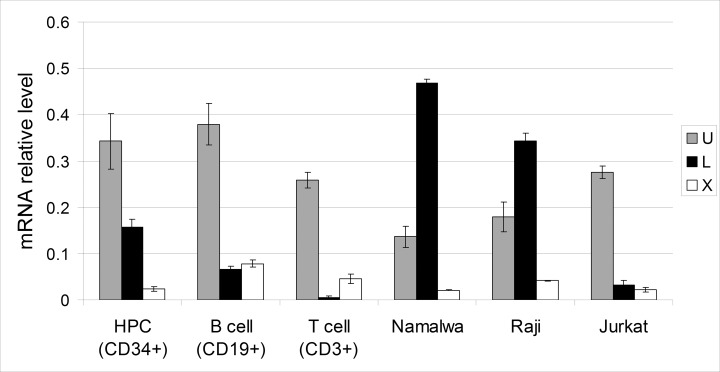
Relative activity of the U, L, and X promoters of the *POU2F1* gene in normal and malignant human hematopoietic cells Transcription levels from L and X promoters were measured by Real-Time PCR. The graphs show means ± S.E.M. for three independent experiments.

To confirm this result, we compared the activity of Oct-1 promoters in normal hematopietic cells and in the tumor cells lines of B- and T-cell origin by qRT-PCR using the corresponding primers (Materials and Methods) (Figure 4). Promoter activity levels were measured in normal hematopoietic cells (progenitor hematopoietic cells (CD34+) and B-cells (CD19+) and T-cells (CD3+) of peripheral blood) and tumor cell lines (Namalwa and Raji B-cell lymphoblastomas and Jurkat T-cell lymphoblastoma).

Our data indicate that the U promoter which regulates ubiquitous Oct-1A transcription is the most active in all the tested normal blood cells. This observation correlates with the high activity of the U promoter in human tissues demonstrated previously [25]. The level of transcription from the X promoter was significantly low compared to that from the U promoter. The X promoter is almost completely repressed in the CD34+ hematopoietic progenitor cells, and starts to function more actively only at the later stages of differentiation in B and T cells. The L promoter which controls the transcription of mRNAs encoding Oct-1L and Oct-1R isoforms functions in the hematopoietic progenitor cells and further retains the high level of its activity only in B-cells.

The activity of the L promoter was also lower than the activity of the U promoter and appeared to be decreased by about two times relative to that of the U promoter at the later stages of B-cell differentiation (compare progenitor hematopoietic cells (CD34+) and (CD19+)). In T cells (CD3+), L promoter activity was dramatically decreased compared to the activity of the U promoter. These results correlate well with the data obtained for the full- length transcripts (Figure 3).

Interestingly, the activity of U promoter was reduced almost two times in the Namalwa and Raji cells compared to the normal B-cells (Figure 4). Significant changes in the L promoter activity were observed in the tumor cells compared to the normal cells. The activity of the L promoter was strongly increased in the Namalwa and Raji B-cell lymphoblastomas. In the Namalwa cells, it became three times higher than the activity of the U promoter. It also showed a significant increase in the Jurkat T-cell lymphoblastoma compared to the normal T-cells (CD3+). Remarkably, all these cell lines were originally obtained from low differentiated lymphoblasts. It seems possible that high levels of Oct-1 isoforms transcribed from the L promoter, in particular of Oct-1L, block the differentiation of B and T-cells at the early stages promoting their malignant transformation. In line with this hypothesis, was the observation that the activity of the X promoter which starts to function at later hematopoetic cell differentiation stages (Figure 3) was lower in these tumor cell lines compared to normal B and T-cells.

In such a way, we have demonstrated that the activity of the alternative promoters of the *POU2F1* gene is significantly altered in the B and T-cell tumor lines compared to the normal B and T-cells.

### Oct-1R acts mainly as a gene transcription repressor and down-regulates the genes responsible for B-cell differentiation and signaling

We aimed next to identify differentially expressed genes (DEGs) which expression is affected by Oct-1R overexpression in the Namalwa B-cell line that was stably transformed with the lentivirus constructs expressing Oct-1R C-terminally fused with 3xFLAG epitope. The amount of transgenic Oct-1R in transformed cells was roughly equal to that of the endogenous Oct-1L (Figure 2C, Namalwa +Oct-1R) indicating that it was expressed at a high, but still physiological level. We analyzed the expression patterns of DEGs by microarray using Illumina HumanHT-12V4 microarrays as it has been done previously for Oct-1A, Oct1L, and Oct-1X isoforms (Pankratova et al, NAR 2016). Changes in the gene transcription level were considered significant with fold change ≥2.0 and Benjamini-Hochberg adjusted *p* ≤ 0.01. Using these criteria we have found 241 DEGs (Supplementary Table 1). The 5′-regulatory regions of most DEGs had the Oct-1 binding site (http://www.genecards.org). For a number of genes, the results of gene expression profiling were confirmed by RT-qPCR (Supplementary Figure 1).

**Figure 5:**
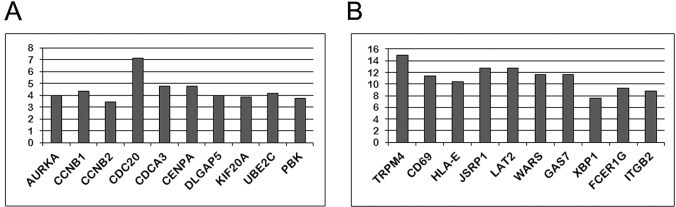
The ten genes most highly up **(A)** and down **(B)** regulated by Oct-1R according to the results of the gene expression analysis using the Illumuna HumanHT-12 microarray. (A) The top ten transcripts up-regulated in the Namalwa cells transformed with the Oct-1R expression construct relative to the control cells (transformed with empty vector). Y axis indicates how many times gene expression increased compared to the control cells. (B) The top ten transcripts down-regulated in the Namalwa cells transformed with the Oct-1R expression construct relative to the control cells (transformed with empty vector). Y axis indicates how many times gene expression decreased compared to the control cells.

Similar to other Oct-1 tissue-specific isoforms, Oct-1R regulated the expression of most Oct-1A target genes, but had its unique targets as well (Supplementary Table 1). Interestingly, overexpression of Oct-1R caused repression (about 80% of identified DEGs) rather than activation of the target genes (20% of identified DEGs). Moreover, the level of transcription repression caused by Oct-1R was generally significantly higher, than the level of transcription activation (compare Figure 5A and 5B). The number of down-regulated genes was significantly higher than it has been found with other isoforms [25].

Similar to what has been found previously for Oct-1A, L, and X isoforms [25], among the ten most strongly up-regulated genes were the genes involved in the cell cycle regulation (Figure 5A). Among the top 10 down-regulated genes were genes encoding proteins responsible for terminal differentiation of B-lymphocytes and immunoglobulin production, namely, CD69, FCER1G, HLA-E, LAT2, TRPM4, ITGB2, XBP1, and GAS7 (Figure 5).

Many of the DEGs identified for the Oct-1R isoform (Table 1) fulfill lymphocyte-specific functions and are involved in the regulation of B-cell differentiation. It is interesting to note that Oct-1R overexpression opposes B-cells differentiation, as far as it leads to the activation of CD24, which modulates B-cell activation response and prevents their terminal differentiation into the antibody-forming cells. We detected the inhibition of genes responsible for the terminal differentiation of B-lymphocytes, immunoglobulin production, and B-cell differentiation in peripheral lymphoid organs, including CD69, FCRL5, LAPTM5, CD70, CD83, ICAM3, XBP1, IFITM1, CR2, FGD2, and MYO1G. The transcription of genes activating B-cell antigen receptor (BCR) mediated signaling (LAT2, BRDG1, STAP1, LAX1, FCRL3) was also found to be down-regulated. This signaling pathway is active in the mature B-cells and is essential for their normal differentiation.

**Table 1:** DEGs involved in B-cell differentiation identified for different Oct-1 isoforms

Gene	Full name	Oct-1A	Oct-1L	Oct-1R	Oct-1X
BRDG1	BCR Downstream Signaling 1	-	-	−3.56	-
BST2	Bone Marrow Stromal Cell Antigen 2	-	−2.71	-	-
CD24	CD24 Antigen	-	-	2.31	2.54
CD27	CD27 Antigen	−3.57	-	-	-
CD48	CD48 Antigen	−2.86	-	−5.57	−3.17
CD52	CD52 Antigen	-	−2.88	-	-
CD53	CD53 Antigen	-	−4.87	−5.07	-
CD69	CD69 Antigen	−5.44	−2.69	−11.44	−4.58
CD70	CD70 Antigen	−2.73	−2.6	−2.48	−2.71
CD83	CD83 Antigen	−4.57	−2.86	−4.01	−3.7
CR2	Complement C3d Receptor 2	−6.99	−4.85	−9.69	−4.41
CTSH	Cathepsin H	-	-	−3.3	-
FCRL3	Fc Receptor Like 3	-	−4.48	−7.2	-
FCRL5	Fc Receptor Like 5	-	-	−5.09	-
FGD2	FYVE, RhoGEF And PH Domain Containing 2	-	−2.65	-	-
HLA-A	Major Histocompatibility Complex, Class I, A	−2.71	−2.98	−3.4	−2.25
HLA-A29.1	Major Histocompatibility Complex, Class I, A	-	−3.29	−4.94	−4.22
HLA-B	Major Histocompatibility Complex, Class I, B	-	−2.86	−3.81	−2.85
HLA-DQA1	Major Histocompatibility Complex, Class II, DQ Alpha 1	-	−4.73	−7	−3.19
HLA-DRB4	Major Histocompatibility Complex, Class II, DR Beta 4	-	−2.87	−2.48	-
HLA-E	Major Histocompatibility Complex, Class I, E	−3.36	−4.65	−10.44	−3.68
HLA-F	Major Histocompatibility Complex, Class I, F	-	−2.7	−3.85	−2.85
HLA-G	Major Histocompatibility Complex, Class I, G	−2.96	−4.23	−5.46	−3.27
HLA-H	Major Histocompatibility Complex, Class I, H	−3.05	−3.27	−3.64	−3.08
ICAM3	Intercellular Adhesion Molecule 3	-	−2.66	−2.76	-
IFI27L2	Interferon Alpha Inducible Protein 27 Like 2	-	−4.15	−3.41	-
IFITM1	Interferon Induced Transmembrane Protein 1	-	−10.16	-	-
ITGB2	Integrin Subunit Beta 2	-	−5.61	−8.06	−2.07
ITM2B	Integral Membrane Protein 2B	-	-	−2.39	-
LAPTM5	Lysosomal Protein Transmembrane 5	-	−2.22	−2.81	-
LAT2	Linker For Activation Of T Cells Family Member 2	−4.0	−5.43	−12.72	−2.58
LAX1	Lymphocyte Transmembrane Adaptor 1	-	-	5.2	-
LTA	Lymphotoxin Alpha	-	-	−4.54	-
MYO1G	Myosin IG	-	−4.84	−7.52	−3.47
STAT2	Signal Transducer And Activator Of Transcription 2	-	-	−4.32	-
TNFRSF14	TNF Receptor Superfamily Member 14	-	−2.92	−3.64	−2.58
TRPM4	Transient Receptor Potential Cation Channel Subfamily M Member 4	−3.48	−7.26	−14.97	−4.99
XBP1	X-Box Binding Protein 1	−2.56	−4.39	−7.38	−3.5

Genes up-regulated or down-regulated more than 2-fold are presented. The spaces indicate that the corresponding genes were neither overexpressed two times or higher, nor down-regulated by 50% in response to Oct-1 overexpression.

At the same time, Oct-1R inhibited genes associated with the differentiation and activation of T-cells and natural killers (NK): CD48, CD53, CD69, CD70, CD27, ITGB2, LAT2, TNFRSF14, LAX1, and TRPM4.

### Differentially regulated genes and processes for Oct-1R are largely similar to the DEGs identified for the Oct-1L isofom

Comparing DEGs for different Oct-1 isoforms identified here and in our previous study, we came to the conclusion that along with controlling a shared set of genes and processes the isoforms also regulate gene pools and pathways which are unique for each of them [25]. The functional analysis of DEGs with DAVID 6.8 demonstrated that even if isoforms are involved in the regulation of the same processes, the number of genes regulated by each of them vary significantly. The pattern of Oct-1R dependent DEGs is very similar to that of Oct-1L. However, 82 specific DEGs were found for Oct-1R compared to Oct-1L indicating that these two isoforms are not functionally identical.

Oct-1L and Oct-1R are the two isoforms which play the most profound role in B-cell development (Table 1). While all Oct-1 isoforms are involved in the control of expression of the B-cell-related genes (Table 1), the highest number of such genes was found for Oct-1R (33 genes) and Oct-1L (28 genes). The lowest number of the genes of this type was found for the ubiquitous Oct-1A isoform (13 genes). Thus, Oct-1L and Oct-1R are the Oct-1 isoforms most deeply involved in B-cell development.

The patterns of B-cell-related DEGs controlled by Oct-1L and Oct-1R overlap considerably (Table 1). Both these isoforms repress the genes responsible for terminal differentiation of B-lymphocytes, immunoglobuline production, and differentiation in peripheral lymphoid organs. Both of them repress the transcription of genes which control BCR-mediated signaling in B-cells. While the expression of some of these genes was influenced by both isoforms, other genes were repressed only by one of them. In such a way, Oct-1R specifically down-regulates BRDG1, FCRL5, ITM2B, LTA, LAX1 and STAP1, while Oct-1L specifically represses several different targets (Table 1).

The analysis has also demonstrated that Oct-1R contributes to the repression of several signaling pathways. Of special interest is the participation of Oct-1R in the repression of IFN type I and IFN-gamma (type II) signaling pathways via the repression of IFNAR2, IFI6, IRF7, IRF9, ISG20, STAT1, STAT2, and the major histocompatibility complex, class I molecules. While all the studied Oct-1 isoforms control certain genes involved in these pathways, Oct-1R was found to play the most important role in their repression (Supplementary Table 2).

Finally, Oct-1R like other Oct-1 isoforms [25] represses the genes involved in the chemokine signaling pathway: CCL3L1, CCL3L3, CCL3, CCL4L1, CCL4L2, FGR, NCF1, STAT1, and STAT2 and cytokine-cytokine receptor interaction: CCL3L1, CCL3L3, CCL3, CCL4L1, CCL4L2, CD70, TNFRSF14, IFNAR2, IL10RB, and LTA. However, in this case, the patterns of genes regulated by different isoforms do not differ significantly.

## DISCUSSION

Although the importance of Oct-1 in cell development and differentiation has been pointed out, the precise roles for this protein as well as the detailed molecular mechanisms of its action are still poorly understood. In the current work, we have proven the existence of the human Oct-1R isoform and characterized its role in gene expression regulation. We have demonstrated that Oct-1R is B-lymphocyte specific, and suggested that its expression pattern is conserved in mammals since the murine Oct-1R homologue is also expressed in B-lymphocytes only [50]. Similar to the Oct-1L-encoding transcript, Oct-1R is transcribed from the L-promoter, but has a shorter open reading frame due to alternative splicing. As a result, Oct-1R isoform lacks 132 C-terminal amino acid residues compared to Oct-1L. The Oct1L isoform is also expressed in B-cells at a high level. However, low levels of Oct1L expression were detected in T-cells as well. We have also demonstrated that Oct-1L level, but not the levels of other Oct-1 isoforms, was considerably increased in Namalwa and Raji B-cell lymphoblastomas compared to the normal B-cells.

As it has been shown previously, Oct-1 is essential for the regulation of the early-stage B-cell and T-cell development and differentiation [57–60]. Our data indicate that several Oct-1 isoforms are expressed in the progenitor hematopoietic cells (CD34+) and in the differentiating B- and T-lymphocytes of peripheral blood. Among these isoforms, only for the ubiquitously expressed Oct-1A isoform the levels of its expression do not differ significantly between the progenitor hematopoietic cells and B- or T-lymphocytes. The expression levels of other isoforms vary depending on the cell type and differentiation stage. For instance, while Oct-1L is expressed in CD34+ cells and B-cells, its level drops dramatically in T-cells. The Oct-1R is B-cell specific and its expression begins in differentiating B-cells. These data also indicate that the L-promoter, which controls the transcription of both isoforms, is specific mostly to B-cells. The Oct-1X isoform is expressed at an extremely low level in HPCs (CD34+). However, its expression level is markedly increased at the later stages of B-cell (CD19+) and T-cell (CD3+) differentiation. Our data indicate that the increase in the Oct-1X expression level in the cells lead to an approximately 10 fold reduction in the LMO2 gene transcription level [25]. LMO2 protein plays a crucial role in hematopoietic development. LMO2 is transcribed at the highest level in the CD34+ progenitor cells and its ectopic overexpression blocks human T-cell development from the CD34+ cells [61, 62].

Our data indicate that the patterns of regulated genes overlap for all studied isoforms, with each isoform controlling also a number of specific genes [25]. In addition, the ability of each isoform to regulate the certain gene varies as well. The data obtained in this study confirm our previous results. We have demonstrated that Oct-1R controls a set of genes shared with other isoforms as well as a set of the isoform-specific genes and acts mainly as a transcription repressor.

The analysis of DEGs in the Namalwa cells transformed with the Oct-1R expressing construct demonstrated that the pattern of genes regulated by Oct-1R is rather similar to that revealed for Oct1L [25]. Both isoforms regulate genes which guide the differentiation of progenitor cells to the B-cell pathway and stabilize the intermediate stage preceding the plasma cells. For example, Oct-1R represses the expression of several genes which govern the differentiation of progenitor cells into other blood cells such as T-cells and NK cells. At the same time, Oct-1R and Oct-1L prevent terminal differentiation of B-cells. For example, they repress the transcription of genes which control the B-cell antigen receptor (BCR) mediated signaling in B-cells, the activity of which is important for normal B-cell differentiation. Oct-1R activates the CD24 gene which modulates the B-cell activation response and prevents terminal differentiation into antibody-forming cells.

Oct-1R also represses the genes which participate in the signal transduction pathways and are responsible for the cell response to certain signals including the chemokine-mediated signaling pathway and cytokine-cytokine receptor signaling. Of special interest is the role of Oct-1R in the repression of the IFN (type I) and IFN-gamma (type II) signaling pathways which are highly important for the immune system differentiation. While all the studied isoforms control various genes involved in these pathways, it is Oct-1R and Oct1L which were found to play the most significant role in their repression.

Our findings indicate that the L-promoter is strongly up-regulated in the tumor B-cell lymphoblastic cell lines Namalwa and Raji, compared to the normal B-cells, while the activity of the U and X promoters is decreased. We have found that the level of the Oct-1L isoform (but not of the Oct-1R isoform, which results from alternative splicing) is strongly increased in these cell lines. In our previous work, we have also observed an increase in the Oct-1L level in several other malignant cell lines [54]. It may be suggested that while Oct-1L is essential for the maintenance of the B-cell pool at the certain stage of development under the normal conditions, the increased levels of Oct-1L in the cell result in the reduced sensitivity of B-cells to the regulatory signals, which suppress proliferation and induce differentiation, and the regulatory interplay between the Oct-1 isoforms contributes to the maintenance of the balance between cell proliferation and cell differentiation. An increase in the activity of the L promoter and a decrease in the X promoter activity were also observed in the Jurkat T-cell line compared to the normal T-lymphocytes.

Oct-1 overexpression was observed in many tumor types [30–35], and Oct-1 may be thus considered a promising therapeutic target in cancer treatment. However, our findings indicate that when using Oct-1 as a therapeutic target it should be kept in mind that Oct-1 is present in the human cells not as a single protein, but as a whole family of functionally different isoforms. Altogether our findings indicate that high level of Oct-1L isoform, but not of other isoforms, may be one of the factors contributing to the malignant transformation of lymphoid cells.

## MATERIALS AND METHODS

### Cell culture and transduction of human cells

B lymphoblastoid Burkitt limphomas Namalwa and Raji and T-cell limphoblastoma Jurkat human cell lines were obtained from the Russian Cell Culture Collection, Institute of Cytology, St. Petersburg, Russia. Cells were maintained in DMEM with 10% FCS, 100 U/mL penicillin, and 100 μg/mL streptomycin. ViraPower Lentiviral Expression System (Invitrogen) was used to obtain stable transduction of cells according to the manufacturer's protocol. Blasticidin was used to maintain the stably transformed cells, and the antibiotic was withdrawn from the media 3 days prior to the experiment. Primary human B-cells, T-cells, and monocytes were obtained from a healthy donor with the informed consent.

### Cell preparation

Peripheral blood mononuclear cells (PBMCs) from healthy donors were prepared by Ficoll density gradient centrifugation. B-cells (CD19+) and T-cells (CD3+) were isolated from PBMCs by positive magnetic selection using the CD19 and CD3 MicroBead Kits, human (Miltenyi Biotec) according to the manufacturer's instructions.

### Expression plasmid construction

The, pL-Oct-1R-3FLAG (C-end) construct was generated by inserting a copy of the human Oct-1R coding sequence into the pLenti6/V5-D-TOPO expression vector (Invitrogen).

### RNA purification, RT-PCR, and RT-qPCR analyses

In the PCR analysis, Human Hematopoietic Progenitor Cells (CD34+) total RNA (Miltenyi Biotec) was used. RNA from primary human hematopoietic cells and cell lines was purified using Trizol. Reverse transcription was performed using the RevertAid H Minus First Strand cDNA Synthesis Kit (Thermo Scientific) and subsequent PCR was carried out using the DyNazyme EXT DNA Polymerase (Biolabs). Primers used are as follows:

Oct-1A A-Forw 5′-tattcaaaatggcggacgga-3′;

Oct-1L, Oct-1R LR-Forw 5′-ccaccccaaactgctacctgt-3′;

Oct-1X X-Forw 5′-cagcacgatttgttggatgtg-3′;

Oct-1Z Z-Forw 5′-atctatggagagtggagatggca-3′;

Oct-1A,L,X,Z Rev-A,L,X,Z 5′-cagtccaatcacactg cagagtg-3′;

Oct-1R Rev-R 5′-ctccacctcagacgtgaatgagat-3′.

qPCR was performed using the qPCRmix-HS-SYBR kit (Evrogen). Primers used are A-Forw, LR-Forw, X-Forw, and Rev 5′-tattcaaaatggcggacgga-3′. mRNA levels were normalized to that of the GUS gene GUS-Forw 5′-cgtggttggagagctcatttgga-3′; GUS-Rev 5′-attccccagcactctcgtcggt-3′. Measurements at each point were made in at least three replicates, and the mean values were calculated.

### Gene expression analysis

Microarrays were performed by Genoanalytica (Moscow, Russian Federation) using the Illumina HumanHT-12V4 Chips (47,300 probes) and the raw microarray data were processed with the aid of the Gene Expression module implemented in the Illumina GenomeStudio Data Analysis Software (version 1.1.1). The *p*-values for differential expression were calculated using the Illumina Custom Error Model algorithm and the Benjamini and Hochberg false discovery rate (FDR), a multiple testing correction method for *p*-value adjustment. Changes in gene transcription were considered significant based when fold change ≥2.0 and Benjamini-Hochberg adjusted *p* ≤0.01. In microarray experiments, intact Namalwa cells and cells transformed with the empty lentivirus were used as controls, with no statistically significant differences having been found between them.

### *In vitro* transcription and translation of Oct-1 isoforms

The Oct-1A/pcDNA3.1, Oct-1L/pcDNA3.1, and Oct-1R/pcDNA3.1 constructs were transcribed and translated in accordance with the TNT^®^ Coupled Reticulocyte Lysate system (Promega) user manual. The synthesized proteins were resolved by 8% SDS–PAGE and detected by Western blot using mouse anti-FLAG antibodies.

### Functional enrichment analysis of DEGs

Gene Ontology (GO) screening was performed using DAVID (david.abcc.ncifcrf.gov/home.jsp) including GOTERM_BP_FAT (biological process), GOTERM_MF_FAT (molecular function), and GOTERM_CC_FAT (cellular component), and KEGG Pathway (www.genome.jp/kegg/pathway.html) resources. DAVID calculates modified Fisher's Exact *p*-values to demonstrate GO or molecular pathway enrichment. The *p*-value <0.01 was chosen as a cut-off criterion.

### Statistical processing

Statistical analysis was performed using Microsoft Excel and Graph Pad Prism. The unpaired Student's t test was used to generate P values. Error bars represent S.E.M. (^*^p < 0.05, and ^**^p < 0.01).

## SUPPLEMENTARY MATERIALS FIGURE AND TABLES





## Abbreviations

POU2F1: POU domain class 2 transcription factor 1
