# Supplementary material for: The regulatory interplay between Oct-1 isoforms contributes to hematopoiesis and the isoforms imbalance correlates with a malignant transformation of B cells
Source: Oncotarget. 2018 Jul 6;9(52):29892–905. doi: 10.18632/oncotarget.25648 (PMC6057458; doi:10.18632/oncotarget.25648)
Supplement: Supplementary file 1 [file oncotarget-09-29892-s001.pdf]

The regulatory interplay between Oct-1 isoforms contributes to hematopoiesis and the isoforms imbalance correlates with a malignant transformation of B cells

SUPPLEMENTARY MATERIALS

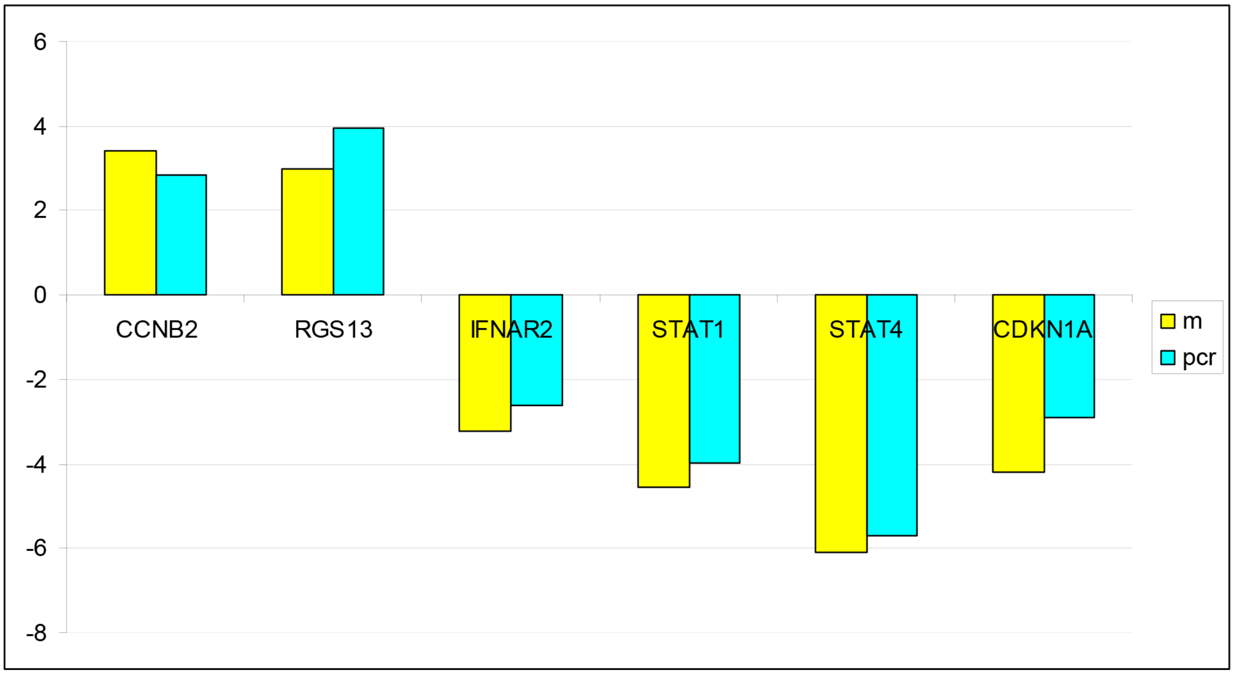

Supplementary Figure 1: qRT-PCR validation of differentially expressed genes from the Namalva cell lines, transfected by Oct-1R isoform. Fold changes determined from the relative Ct values of the SybrGreen Gene Expression assay for CCNB2, RGS13, IFNAR2, STAT1, STAT4 and CDKN1A genes were compared to those detected by RNA-microarray. Replicates (n=3) of each sample were run and the Ct values were normalized to 18S RNA. For validation of the microarray results, genes from the gene list were selected for qRT-PCR analysis.

Supplementary Table 1: Fold change for DEGs in the case of Oct-1R overexpression in Namalwa cells.

See Supplementary File 1

Supplementary Table 2: Fold change of DEGs related to Type I interferon signaling pathway

Gene	Full name	Oct-1R	Oct-1L	Oct-1A	Oct-1X
BST2	bone marrow stromal cell antigen 2	-	-2.71	-	-
ISG15	ISG15 ubiquitin-like modifier	-5.48	-4.75	-3.95	-2.71
MX1	MX dynamin like GTPase 1	-2.77	-	-	-
EGR1	early growth response 1(EGR1)	-3.81	-3.16	-3.73	-3.66
GBP2	guanylate binding protein 2	-	-	-5.51	-10.2
IFNAR2	interferon alpha and beta receptor subunit 2	-3.23	-3.05	-2.91	-5.48
IFI6	interferon alpha inducible protein 6	-4.58	-4.04	-	-2.63
IRF7	interferon regulatory factor 7	-5.17	-3.62	-3.74	-5.43
IRF9	interferon regulatory factor 9	-3.76	-	-	-2.72
ISG20	interferon stimulated exonuclease gene 20	-3.12	-2.55	-2.49	-2.71
HLA-A	major histocompatibility complex, class I, A	-3.4	-2.98	-2.71	-2.25
HLA-B	major histocompatibility complex, class I, B	-3.81	-2.86	-	-2.85
HLA-E	major histocompatibility complex, class I, E	-10.44	-4.65	-3.36	-3.68
HLA-F	major histocompatibility complex, class I, F	-3.85	-2.7	-	-2.85
HLA-G	major histocompatibility complex, class I, G	-5.46	-4.23	-2.96	-3.27
HLA-H	major histocompatibility complex, class I, H	-3.64	-3.27	-3.05	-3.08
STAT1	signal transducer and activator of transcription 1	-5.49	-2.67	-2.94	-5.08
STAT2	signal transducer and activator of transcription 2	-4.32	-	-	-

Genes up-regulated or down-regulated more than 2-fold are presented. The spaces indicate that the corresponding genes were neither overexpressed two times or more nor down-regulated by 50% in response to Oct-1 overexpression.
