# Supplementary material for: The regulatory interplay between Oct-1 isoforms contributes to hematopoiesis and the isoforms imbalance correlates with a malignant transformation of B cells
Source: Oncotarget. 2018 Jul 6;9(52):29892–905. doi: 10.18632/oncotarget.25648 (PMC6057458; doi:10.18632/oncotarget.25648)
Supplement: Supplementary file 2 [file oncotarget-09-29892-s002.docx]

**Supplementary Table 1: Fold change for DEGs in the case of Oct-1R overexpression in Namalwa cells**

| Target_ID | N.Diff Pval | PROTEIN_PRODUCT | PROBE_ID | Fold Change Ratio N/N1R |
| --- | --- | --- | --- | --- |
| ABCB9 | 0.00168 | NP_062570.1 | ILMN_2343047 | 3.51730959 |
| ABCB9 | 0.00017 | NP_062570.1 | ILMN_2343048 | 3.47546368 |
| ACAP1 | 0.00912 | NP_055531.1 | ILMN_1808395 | 3.22801932 |
| ALDOC | 0.00001 | NP_005156.1 | ILMN_1755974 | 3.31922088 |
| AP3B1 | 0 | NP_003655.3 | ILMN_1768867 | 0.26576013 |
| ASNS | 0.00001 | NP_597680.1 | ILMN_1796417 | 4.4148398 |
| ASNS | 0 | NP_597680.1 | ILMN_2398107 | 4.67806841 |
| ATF5 | 0.00078 | NP_036200.2 | ILMN_1669113 | 3.47428623 |
| ATP6V0A1 | 0.00007 | NP_005168.2 | ILMN_1752579 | 2.86036036 |
| AURKA | 0.00159 | NP_940838.1 | ILMN_1680955 | 0.25492302 |
| AURKA | 0.00719 | NP_940836.1 | ILMN_2357438 | 0.25394785 |
| AURKB | 0.00519 | NP_004208.2 | ILMN_1684217 | 0.32077512 |
| AXUD1 | 0.00123 | NP_149016.1 | ILMN_1703123 | 3.97906699 |
| BIRC3 | 0.00409 | NP_892007.1 | ILMN_2405684 | 2.33739775 |
| BIRC3 | 0.00005 | NP_001156.1 | ILMN_1776181 | 3.8721715 |
| BRDG1 | 0.00508 | NP_036240.1 | ILMN_1781085 | 3.56806283 |
| BZW2 | 0.00139 | NP_054757.1 | ILMN_1676548 | 0.32432866 |
| C10ORF75 | 0.00028 |  | ILMN_3268880 | 6.14907652 |
| C12ORF48 | 0.00047 | NP_060385.2 | ILMN_1727055 | 0.31228493 |
| C20ORF100 | 0.00039 | NP_116272.1 | ILMN_2082209 | 5.22256729 |
| C20ORF199 | 0.00131 |  | ILMN_3188984 | 2.82694269 |
| CAMK2N1 | 0.00024 | NP_061054.2 | ILMN_1794863 | 3.76005679 |
| CASP1 | 0.00544 | NP_150636.1 | ILMN_2326512 | 5.03966942 |
| CBLN3 | 0.00138 | NP_001034860.1 | ILMN_2053829 | 3.72810358 |
| CCDC85A | 0.00347 | NP_001073902.1 | ILMN_1669982 | 3.49957663 |
| CCL3 | 0 | NP_002974.1 | ILMN_1671509 | 6.8686606 |
| CCL3L1 | 0.00001 | NP_066286.1 | ILMN_2218856 | 5.83980182 |
| CCL3L1 | 0 | NP_066286.1 | ILMN_1747355 | 5.66073457 |
| CCL3L3 | 0 | NP_001001437.2 | ILMN_2105573 | 5.2715688 |
| CCL4L1 | 0 | NP_001001435.1 | ILMN_2100209 | 3.61652719 |
| CCL4L2 | 0 | NP_996890.1 | ILMN_1716276 | 4.13687861 |
| CCNB1 | 0.00012 | NP_114172.1 | ILMN_1712803 | 0.23829624 |
| CCNB2 | 0.00365 | NP_004692.1 | ILMN_1801939 | 0.2971351 |
| CD24 | 0.00696 | NP_037362.1 | ILMN_2060413 | 0.45659869 |
| CD48 | 0.00005 | NP_001769.2 | ILMN_2061043 | 5.57879437 |
| CD53 | 0.00012 | NP_000551.1 | ILMN_2413808 | 5.07103825 |
| CD53 | 0.00001 | NP_000551.1 | ILMN_1662843 | 5.78912685 |
| CD69 | 0 | NP_001772.1 | ILMN_2188333 | 11.4494609 |
| CD70 | 0.00835 | NP_001243.1 | ILMN_1760247 | 2.48027864 |
| CD83 | 0.00043 | NP_001035370.1 | ILMN_1780582 | 4.01214182 |
| CD83 | 0.00142 | NP_004224.1 | ILMN_2328666 | 5.14078675 |
| CDC20 | 0 | NP_001246.2 | ILMN_1663390 | 0.14663794 |
| CDCA3 | 0.00159 | NP_112589.1 | ILMN_1737728 | 0.21677717 |
| CDCA8 | 0.00098 | NP_060571.1 | ILMN_1709294 | 0.28621241 |
| CDKN1A | 0.00168 | NP_000380.1 | ILMN_1784602 | 2.89331268 |
| CENPA | 0.00027 | NP_001035891.1 | ILMN_1801257 | 0.21778697 |
| CENPV | 0.00154 | NP_859067.2 | ILMN_1729142 | 0.30489744 |
| CHEK1 | 0.00396 | NP_001265.1 | ILMN_1664630 | 0.2748138 |
| COPB1 | 0.00925 | NP_057535.1 | ILMN_1699112 | 2.38512988 |
| COX7A2L | 0.00434 | NP_004709.2 | ILMN_3237665 | 2.41715511 |
| CR2 | 0.00016 | NP_001868.2 | ILMN_2369666 | 9.69905213 |
| CREB3L2 | 0.00039 | NP_919047.2 | ILMN_1751097 | 2.88461538 |
| CST3 | 0.00198 | NP_000090.1 | ILMN_1800354 | 4.88825623 |
| CTSH | 0.00697 | NP_004381.2 | ILMN_2390853 | 3.30873699 |
| DBNDD1 | 0 | NP_001036075.1 | ILMN_2374352 | 7.13875069 |
| DCTPP1 | 0.0058 | NP_077001.1 | ILMN_3242459 | 0.33135113 |
| DCTPP1 | 0.00207 | NP_077001.1 | ILMN_1763129 | 0.33786049 |
| DDX10 | 0.00835 | NP_004389.2 | ILMN_1753249 | 0.37831955 |
| DIMT1L | 0.00737 | NP_055288.1 | ILMN_1803312 | 0.4635844 |
| DLGAP5 | 0.00049 | NP_055565.2 | ILMN_1749829 | 0.25759577 |
| DNASE2 | 0.00006 | NP_001366.1 | ILMN_1796245 | 8.33668342 |
| DUSP22 | 0.00511 | NP_064570.1 | ILMN_1730765 | 4.94974874 |
| DUSP22 | 0 | NP_064570.1 | ILMN_1671809 | 7.17474371 |
| DUSP22 | 0 | XP_946784.1 | ILMN_1813275 | 4.82367965 |
| DUSP5 | 0.00001 | NP_004410.3 | ILMN_1656501 | 7.75079114 |
| DUT | 0.00213 | NP_001020419.1 | ILMN_1732688 | 0.38884709 |
| EBNA1BP2 | 0.00409 | NP_006815.1 | ILMN_1768127 | 0.36859275 |
| EDEM1 | 0.00549 | NP_055489.1 | ILMN_1779828 | 2.60828919 |
| EEF1B2 | 0.00139 | NP_066944.1 | ILMN_1685678 | 0.32090546 |
| EGR1 | 0.00193 | NP_001955.1 | ILMN_1762899 | 3.81047382 |
| FABP5 | 0.00204 | NP_001435.1 | ILMN_2146761 | 0.27242421 |
| FABP5 | 0.00062 | NP_001435.1 | ILMN_1696302 | 0.2736441 |
| FABP5L2 | 0.00744 | XP_001721224.1 | ILMN_3178258 | 0.30760479 |
| FAM116B | 0.00009 | NP_001001794.2 | ILMN_1659029 | 4.10459492 |
| FCER1G | 0.00001 | NP_004097.1 | ILMN_2123743 | 9.32258065 |
| FCRL3 | 0.00016 | NP_001019838.1 | ILMN_1691693 | 7.20910624 |
| FCRL5 | 0.00874 | NP_112571.1 | ILMN_1684445 | 5.09711684 |
| FGR | 0 | NP_005239.1 | ILMN_1795158 | 7.90522737 |
| FGR | 0.00001 | NP_001036194.1 | ILMN_2368318 | 6.42960725 |
| FLJ35024 | 0.0091 |  | ILMN_3243324 | 4.73521851 |
| FNBP1 | 0.00013 | NP_055848.1 | ILMN_1797342 | 3.33151927 |
| FNDC3A | 0.0004 | NP_001073141.1 | ILMN_2362581 | 5.54026846 |
| FSCN1 | 0.0011 | NP_003079.1 | ILMN_1808707 | 2.83071517 |
| FYN | 0.00279 | NP_694592.1 | ILMN_1686555 | 3.8869382 |
| GAS7 | 0.00004 | NP_958839.1 | ILMN_1745994 | 11.6781857 |
| GLG1 | 0.00001 | NP_036333.2 | ILMN_1772261 | 3.54178165 |
| GRN | 0.00396 | NP_002078.1 | ILMN_1811702 | 3.95550162 |
| GSTA4 | 0 | NP_001503.1 | ILMN_1771964 | 7.26418787 |
| GTF3C6 | 0.00952 | NP_612417.1 | ILMN_1691578 | 0.3914196 |
| H1F0 | 0.00374 | NP_005309.1 | ILMN_1757467 | 4.27980296 |
| HCP5 | 0 | NP_006665.2 | ILMN_1803945 | 8.46544546 |
| HCST | 0.00004 | NP_001007470.1 | ILMN_1699931 | 4.04590892 |
| HDGF | 0.00279 | NP_004485.1 | ILMN_1765621 | 0.4178365 |
| HERPUD1 | 0.00111 | NP_001010990.1 | ILMN_2374159 | 2.96264718 |
| HERPUD1 | 0.00095 | NP_001010990.1 | ILMN_2374164 | 2.56695906 |
| HLA-A | 0.00008 | NP_002107.3 | ILMN_1671054 | 3.4052278 |
| HLA-A | 0 | NP_002107.3 | ILMN_2203950 | 3.46238225 |
| HLA-A29.1 | 0.00003 | NP_001074309.1 | ILMN_2165753 | 4.94921191 |
| HLA-B | 0.00003 | NP_005505.2 | ILMN_1778401 | 3.81673787 |
| HLA-DQA1 | 0.00004 | XP_941221.1 | ILMN_1808405 | 7.00788177 |
| HLA-DRB4 | 0.00697 | NP_068818.4 | ILMN_1752592 | 2.48607337 |
| HLA-E | 0 | NP_005507.3 | ILMN_1765258 | 10.4473282 |
| HLA-F | 0.00011 | NP_061823.1 | ILMN_2186806 | 3.85923725 |
| HLA-G | 0.00001 | NP_002118.1 | ILMN_1656670 | 5.46807229 |
| HLA-H | 0.00457 |  | ILMN_1666078 | 3.64917197 |
| HLA-H | 0.00005 |  | ILMN_2130441 | 3.99347598 |
| HMGB1L1 | 0.00011 | NP_001008735.1 | ILMN_1809439 | 0.3133385 |
| HS.143018 | 0.00222 |  | ILMN_1824362 | 3.53070175 |
| HS.193767 | 0.00166 |  | ILMN_1903914 | 4.17853348 |
| HS.5724 | 0 |  | ILMN_1839019 | 6.84749801 |
| HSPA1B | 0.00732 | NP_005337.1 | ILMN_1660436 | 0.42766414 |
| HSPA6 | 0.00194 | NP_002146.2 | ILMN_1806165 | 6.1032413 |
| ICAM3 | 0.00027 | NP_002153.1 | ILMN_2212763 | 2.76430892 |
| IFI27L2 | 0.0038 | NP_114425.1 | ILMN_1740319 | 3.4189294 |
| IFI6 | 0.00003 | NP_075011.1 | ILMN_1687384 | 4.58615611 |
| IFNAR2 | 0.00026 | NP_997467.1 | ILMN_1765146 | 3.23906409 |
| IL10RB | 0.00001 | NP_000619.3 | ILMN_2230892 | 2.99681106 |
| IRF7 | 0.00012 | NP_004020.1 | ILMN_1798181 | 5.17209302 |
| IRF9 | 0.00065 | NP_006075.3 | ILMN_1745471 | 3.76550388 |
| ISG15 | 0.00001 | NP_005092.1 | ILMN_2054019 | 5.48498062 |
| ISG20 | 0.0006 | NP_002192.2 | ILMN_1659913 | 3.12722803 |
| ITGB2 | 0 | NP_000202.2 | ILMN_2175912 | 8.06244027 |
| ITGB2 | 0 | NP_000202.1 | ILMN_1654396 | 8.74105263 |
| ITGB7 | 0.00016 | NP_000880.1 | ILMN_1777519 | 5.06139438 |
| ITM2B | 0.00331 | NP_068839.1 | ILMN_1751708 | 2.3937188 |
| JSRP1 | 0 | NP_653217.1 | ILMN_1739726 | 12.7591241 |
| KIF20A | 0.00166 | NP_005724.1 | ILMN_1695658 | 0.25393733 |
| LAPTM5 | 0.00039 | NP_006753.1 | ILMN_1772359 | 2.81254125 |
| LAT2 | 0.00001 | NP_115853.2 | ILMN_2326953 | 12.7244259 |
| LAT2 | 0 | NP_071323.1 | ILMN_1803560 | 11.0396624 |
| LAX1 | 0.00469 | NP_060243.2 | ILMN_1769782 | 5.20890411 |
| LMTK3 | 0.00367 | XP_941465.1 | ILMN_1668194 | 3.26787981 |
| LOC100129681 | 0.00123 | XP_001726886.1 | ILMN_3259146 | 2.441385 |
| LOC100130070 | 0.00459 | XP_001723941.1 | ILMN_3264073 | 2.32421113 |
| LOC100130561 | 0.0009 | XP_001723241.1 | ILMN_3265797 | 0.37394439 |
| LOC100131572 | 0.00379 | XP_001725235.1 | ILMN_3289171 | 2.45670442 |
| LOC100131787 | 0.00085 |  | ILMN_3199974 | 2.45211742 |
| LOC100133329 | 0.00299 |  | ILMN_3235357 | 2.82218766 |
| LOC100133583 | 0.00024 | XP_001714126.1 | ILMN_3214389 | 4.85699374 |
| LOC100133678 | 0 | XP_001719856.1 | ILMN_3249667 | 7.30775564 |
| LOC100134291 | 0.00062 | XP_001718122.1 | ILMN_3249560 | 6.76202694 |
| LOC148915 | 0.00329 | XP_942851.1 | ILMN_1776052 | 0.36883902 |
| LOC387934 | 0.00036 | XP_942601.1 | ILMN_1779852 | 0.33669517 |
| LOC642073 | 0.00302 |  | ILMN_3243714 | 2.65733673 |
| LOC642956 | 0.00004 | XP_943259.1 | ILMN_3210741 | 0.25403034 |
| LOC649143 | 0.00489 | XP_949915.1 | ILMN_1815895 | 2.9860661 |
| LOC651816 | 0.00463 | XP_946153.1 | ILMN_1729115 | 0.42946612 |
| LOC728835 | 0 | XP_001133190.1 | ILMN_3235832 | 5.41168659 |
| LOC728877 | 0.00111 |  | ILMN_3244395 | 0.41781342 |
| LOC729255 | 0.0004 |  | ILMN_3227563 | 2.67922078 |
| LOC729779 | 0.00002 |  | ILMN_3305273 | 4.04109589 |
| LOC730278 | 0.00004 | XP_001126471.1 | ILMN_3241234 | 3.52921865 |
| LOC731682 | 0.00467 | XP_001129369.1 | ILMN_1791534 | 5.03790087 |
| LPP | 0 | NP_005569.1 | ILMN_1651254 | 6.75574185 |
| LTA | 0.00974 | NP_000586.2 | ILMN_1795464 | 4.54907162 |
| LY96 | 0.00006 | NP_056179.1 | ILMN_1724533 | 4.1701364 |
| MAP1LC3B | 0.0059 | NP_073729.1 | ILMN_1703244 | 2.7887413 |
| MARCKS | 0.0001 | NP_002347.5 | ILMN_1807042 | 3.16266667 |
| MCL1 | 0.0051 | NP_068779.1 | ILMN_1803988 | 2.36088496 |
| MCM2 | 0.00469 | NP_004517.2 | ILMN_1681503 | 0.3678081 |
| METTL1 | 0.00074 | NP_005362.3 | ILMN_3306997 | 0.26294166 |
| MIR155HG | 0 |  | ILMN_3248910 | 7.48202614 |
| MX1 | 0.00016 | NP_002453.1 | ILMN_1662358 | 2.77998368 |
| MXD4 | 0.00367 | NP_006445.1 | ILMN_1756541 | 3.01364437 |
| MYO1G | 0.00002 | NP_149043.1 | ILMN_1692295 | 7.52008929 |
| NCF1 | 0.0001 | NP_000256.3 | ILMN_1697309 | 4.31431465 |
| NCF1C | 0.00015 |  | ILMN_2112988 | 4.02264808 |
| NOP56 | 0.00289 | NP_006383.2 | ILMN_1705407 | 0.35725235 |
| NOP56 | 0.0017 | NP_006383.2 | ILMN_2044832 | 0.38702794 |
| NPPA | 0.00042 | NP_006163.1 | ILMN_1750386 | 3.90316206 |
| ODC1 | 0.00045 | NP_002530.1 | ILMN_1748591 | 0.31053361 |
| P2RX1 | 0.006 | NP_002549.1 | ILMN_1758529 | 6.57981221 |
| PBK | 0.00409 | NP_060962.2 | ILMN_1673673 | 0.2771609 |
| PCK2 | 0 | NP_004554.2 | ILMN_1671791 | 5.94708382 |
| PFKFB4 | 0.00442 | NP_004558.1 | ILMN_1653292 | 3.47518126 |
| PIM2 | 0.00139 | NP_006866.2 | ILMN_1748283 | 2.98367654 |
| PLAC8 | 0.002 | NP_057703.1 | ILMN_2093343 | 2.41040671 |
| PLAC8 | 0.00025 | NP_057703.1 | ILMN_1653026 | 3.0375438 |
| PLEK | 0 | NP_002655.1 | ILMN_1795762 | 5.65053481 |
| PPIH | 0.00168 | NP_006338.1 | ILMN_1801913 | 0.35322701 |
| PPP1R15A | 0.00045 | NP_055145.2 | ILMN_1659936 | 3.89530042 |
| PRNP | 0.00697 | NP_001073590.1 | ILMN_1737988 | 3.09367246 |
| PSAP | 0.00076 | NP_002769.1 | ILMN_2355559 | 2.59642401 |
| PSAT1 | 0.00003 | NP_066977.1 | ILMN_1692938 | 3.44038908 |
| QPRT | 0.00671 | NP_055113.2 | ILMN_1700268 | 2.66890697 |
| RAB11FIP1 | 0.00286 | NP_001002814.1 | ILMN_1692219 | 2.42317557 |
| RASGRP3 | 0.00007 | NP_733772.1 | ILMN_1727045 | 3.17344647 |
| RASSF6 | 0.00003 | NP_958834.1 | ILMN_1745820 | 4.20884956 |
| RGS13 | 0.00266 | NP_658912.1 | ILMN_2407775 | 0.34424824 |
| RICH2 | 0.00043 | NP_055674.4 | ILMN_3245564 | 6.35062612 |
| RIMS3 | 0.00311 | NP_055562.2 | ILMN_1742382 | 3.11992168 |
| RNASET2 | 0 | NP_003721.2 | ILMN_1671565 | 7.25807265 |
| RNU1A3 | 0.00006 |  | ILMN_3245678 | 3.71328321 |
| RNU4-2 | 0.00995 |  | ILMN_3308138 | 4.50882825 |
| RPL15 | 0.00159 | NP_002939.2 | ILMN_1762747 | 2.64007652 |
| RTN3 | 0.00952 | NP_006045.1 | ILMN_2320906 | 3.63417306 |
| S100A11 | 0.00367 | NP_005611.1 | ILMN_1750101 | 3.29284426 |
| SC4MOL | 0.00974 | NP_001017369.1 | ILMN_1720889 | 2.45358159 |
| SCARNA9 | 0.00729 |  | ILMN_1805064 | 3.34383033 |
| SEMA4A | 0.00001 | NP_071762.2 | ILMN_1702787 | 5.92977191 |
| SLC7A7 | 0.00325 | NP_003973.2 | ILMN_1810275 | 6.79310345 |
| SMPDL3A | 0.00648 | NP_006705.1 | ILMN_1796349 | 5.33773585 |
| SRGN | 0 | NP_002718.2 | ILMN_2169152 | 4.79223744 |
| SRGN | 0 | NP_002718.2 | ILMN_1760347 | 5.07004705 |
| STAP1 | 0.00697 | NP_036240.1 | ILMN_3247998 | 3.48407643 |
| STAT1 | 0 | NP_644671.1 | ILMN_1691364 | 5.49163275 |
| STAT1 | 0 | NP_009330.1 | ILMN_1777325 | 3.9880015 |
| STAT2 | 0.00034 | NP_005410.1 | ILMN_1690921 | 4.32727273 |
| STAT4 | 0.00048 | NP_003142.1 | ILMN_1785202 | 6.0915493 |
| STC2 | 0.00019 | NP_003705.1 | ILMN_1691884 | 5.44007491 |
| STYXL1 | 0.00572 | NP_057170.1 | ILMN_2210729 | 2.41307815 |
| SUSD3 | 0.00699 | NP_659443.1 | ILMN_1785570 | 3.02487884 |
| TCEA1 | 0.00017 | NP_958845.1 | ILMN_2357770 | 4.0738718 |
| TESC | 0.00026 | NP_060369.2 | ILMN_1750181 | 3.43396226 |
| TMEM154 | 0.00001 | NP_689893.1 | ILMN_2088124 | 5.24700071 |
| TNFRSF14 | 0.00024 | NP_003811.2 | ILMN_1697409 | 3.64795059 |
| TP53INP1 | 0.00341 | NP_150601.1 | ILMN_2214197 | 3.82924901 |
| TP63 | 0.00048 | NP_001108453.1 | ILMN_3305055 | 3.67532468 |
| TPP1 | 0.0064 | NP_000382.3 | ILMN_1729234 | 3.20210665 |
| TRIB3 | 0.00007 | NP_066981.2 | ILMN_1787815 | 4.05096419 |
| TRIP13 | 0.00299 | NP_004228.1 | ILMN_1796589 | 0.32133415 |
| TRMT5 | 0.00952 | NP_065861.1 | ILMN_2167922 | 0.40274231 |
| TRPM4 | 0 | NP_060106.2 | ILMN_1679401 | 14.9788839 |
| TSC22D3 | 0.00004 | NP_932174.1 | ILMN_1748124 | 5.85822511 |
| TSPAN8 | 0.00022 | NP_004607.1 | ILMN_1683263 | 5.45314353 |
| TYSND1 | 0.00213 | NP_001035363.1 | ILMN_1775677 | 0.36341441 |
| UBE2C | 0.00027 | NP_861516.1 | ILMN_2301083 | 0.28714755 |
| UBE2C | 0.00017 | NP_861519.1 | ILMN_1714730 | 0.24140114 |
| UBE2H | 0.00366 | NP_003335.1 | ILMN_1757644 | 3.9877551 |
| UCHL5IP | 0.004 | NP_059988.3 | ILMN_2371700 | 0.39532505 |
| UHRF1 | 0.00689 | NP_001041666.1 | ILMN_1786065 | 0.40513555 |
| WARS | 0 | NP_776049.1 | ILMN_1727271 | 11.6776333 |
| WARS | 0.00002 | NP_004175.2 | ILMN_2337655 | 7.61761488 |
| WDR12 | 0.00434 | NP_060726.2 | ILMN_2045729 | 0.40616016 |
| WDR74 | 0.0051 | XP_001125771.1 | ILMN_1789775 | 0.30404601 |
| XBP1 | 0 | NP_001073007.1 | ILMN_2365465 | 7.38748446 |
| XBP1 | 0 | NP_005071.2 | ILMN_1809433 | 7.515163 |
| YIPF1 | 0.00007 | NP_061855.1 | ILMN_2052163 | 3.42397454 |
| ZFP36 | 0.00024 | NP_003398.1 | ILMN_1720829 | 3.10307692 |
| ZNF275 | 0.0013 | NP_001073954.1 | ILMN_2243553 | 4.07647059 |

Cells were stably transformed with the construct expressing Oct-1R.

Genes up-regulated or down-regulated more than 2-fold are presented.
